# *Lactobacillus paracasei* Reduces Intestinal Inflammation in Adoptive Transfer Mouse Model of Experimental Colitis

**DOI:** 10.1155/2011/807483

**Published:** 2011-07-25

**Authors:** Manuel Oliveira, Nabil Bosco, Genevieve Perruisseau, Jeanne Nicolas, Iris Segura-Roggero, Stéphane Duboux, Muriel Briand, Stéphanie Blum, Jalil Benyacoub

**Affiliations:** ^1^Nutrition and Health Department, Nestlé Research Center, CH 1000 Lausanne, Switzerland; ^2^Food Immunology Group, Vers-chez-les-Blanc, CH 1000 Lausanne 26, Switzerland; ^3^Bioanalytical Sciences Department, Nestlé Research Center, CH 1000 Lausanne, Switzerland

## Abstract

Studies showed that specific probiotics provide therapeutic benefits in inflammatory bowel disease. *In vitro* evidence suggested that *Lactobacillus paracasei* also called ST11 (CNCM I-2116) is a potent strain with immune modulation properties. However, little is known about its capacity to alleviate inflammatory symptoms *in vivo* In this context, the main objective of this study was to investigate the role of ST11 on intestinal inflammation using the adoptive transfer mouse model of experimental colitis. Rag2^−/−^ recipient mice were fed with ST11 (10^9^ CFU/day)a month prior toinduce colitis by adoptive transfer of naive T cells. One month later, in clear contrast to nonfed mice, weight loss was significantly reduced by 50% in ST11-fed mice. Further analysis of colon specimens revealed a significant reduction neutrophil infiltration and mucosal expression of IL1*β*, IL-6, and IL12 proinflammatory cytokines, whereas no consistent differences in expression of antibacterial peptides or tight junction proteins were observed between PBS and ST11-fed mice. All together, our results demonstrate that oral administration of ST11 was safe and had a significant preventive effect on colitis. We conclude that probiotics such as *Lactobacillus paracasei* harbor worthwhile *in vivo* immunomodulatory properties to prevent intestinal inflammation by nutritional approaches.

## 1. Introduction

Probiotics are defined as live microbial food supplements that when ingested can survive gastrointestinal tract and exert positive influence on host health. The mode of action of probiotics is complex and not yet fully elucidated. Many mechanisms have been reported to explain probiotic actions such as antagonism against intestinal pathogens, enhancement of mucosal barrier activity, or modulation of host's immune functions as recently reviewed in [1].

Inflammatory bowel disease (IBD) is a term used to cover a large range of immune-mediated diseases with not well-defined aetiology that results in chronic relapsing inflammation of the gut. The two major forms of IBD are Crohn's disease and ulcerative colitis. Genetic predispositions as well as environmental factors such as diet or composition and activity of intestinal microbiota have been implicated in IBD pathogenesis [2]. Experimental colitis induced by adoptive transfer (ECIBAT) of naïve T cells in lymphopenic mice is an established animal model for IBD sharing a number of clinical, genetic, and immunological features with the human disease [3, 4]. Thus, ECIBAT is considered as one of the most relevant models to study IBD pathogenesis or to design and evaluate therapies. 

In rodents, different probiotic cocktails (some are already commercially available) were effective in preventing or reducing gut inflammation when administrated before inducing intestinal injury. For instance, considerable benefits in animals fed with a combination of lactic acid-producing bacteria (LAB) were reported with* Lactobacillus salivarius* and *Bifidobacterium infantis,* YO-MIX Y 109 FRO (3 strains of LAB), IRT5 (5 strains of LAB), or VSL#3 (8 strains of LAB) [5–13]. Some probiotic feeding protocol significantly reduced intestinal disease severity with weight loss reduction and or improvement of colon pathology over the experimental period [1, 14–16]. However, the clinical studies with IBD patients fed with the same probiotic cocktails are either missing or did not systematically and consistently induce clinical remission. The studies made so far underline the need to further study and understand IBD in order to optimize the potential nutritional solution to ameliorate IBD. 

*Lactobacillus paracasei *ST11 (NCC2461) was previously shown to adhere to intestinal epithelial cell line and have antimicrobial activity *in vitro* [17–19]. It was also shown that ST11 decreases nonrotavirus diarrhea in infants [19, 20]. We also observed that daily intake of ST11 tends to interfere with *Helicobacter pylori* colonization in healthy infants and adults [21, 22]. ST11 strains provide convincing and interesting health benefits associated with gastrointestinal tract physiology, however, no evidences exist concerning potential protection against intestinal inflammation. Herein, the main objectives of this work were to complete our knowledge on ST11 *in vitro* properties and to evaluate the protective properties of ST11 in a mouse model of ECIBAT.

## 2. Materials and Methods

### 2.1. Animals

Wild-type (WT) or Rag2^−/−^ C57BL/6 mice were purchased from CDTA Orleans (France). Mice were maintained in specific pathogen-free conditions at Nestlé Research Center animal care facility. Female mice were used around 7 weeks of age and ST11-fed for the next 8 weeks (4 weeks pre- and postcolitis induction) as described below. All experiments were conducted according to the Nestlé Research Center use and care of experimental animal committee and approved by Swiss governmental veterinary offices (authorisation number VD2076). All animal displaying signs of pain or >10% weight loss have to be prematurely killed.

### 2.2. Probiotic Bacteria Culture, Administration, and Detection

*Lactobacillus paracasei* ST11 (NCC2461) bacteria were grown in MRS broth at 37°C for 16–18 h, and then number of viable cells was determined by agar plate counting and/or OD_600_ measurements. For *in vitro* experiments, fresh cultures were used, whereas ST11 bacterial stocks were made in PBS with 10% glycerol and kept frozen at −80°C until used for *in vivo* experiments. Each day a vial was thawed, extensively washed, and resuspended in PBS before administration by gavage to each animal. ST11-fed animals received 10^9^ CFU of live bacteria daily in 200 *μ*L PBS, whereas control received only PBS. Mice were fed from the beginning until the end of the study.

### 2.3. Bone Marrow-Derived Dendritic Cell Culture and Stimulation

C57BL/6 bone marrow-derived dendritic cells (BM-DCs) were differentiated *in vitro* using previously described protocol [23]. BM-DC were harvested, washed, and counted for stimulation after 5–7 days culture in Iscove's modified Dulbecco medium (IMDM) supplemented with 10% heat-inactivated fetal calf serum, 100 U/mL penicillin, 100 *μ*g/mL streptomycin, 5 mM *β*-mercaptoethanol (all Sigma), and 10 ng/mL of human recombinant Fms-like tyrosine kinase 3 ligand (FLT3L, R&D systems). We obtained in routine >95% of immature CD11c^+^ BM-DC as estimated by flow cytometry analysis (FACS Calibur, Becton Dickinson) of live-gated cells. BM-DCs were matured by 24 hours incubation with ST11, lipopolysaccharide (LPS, *E.coli* 055:B5, Sigma), or lipotechoïc acid (LTA, *S. aureus*, Sigma) at indicated concentrations. BM-DC phenotypic maturation was assessed by anti-CD40, -CD80, and -CD86 staining and flow cytometry analysis of live CD11b^+^CD11c^+^cells. All antibodies were purchased from eBiosciences. Additionally, as an indication of BM-DC functional activation, proinflammatory cytokine expression was assessed by ELISA as described below.

### 2.4. T Helper Cell Differentiation Assay

CD4^+^ T cells were isolated from spleen and lymph nodes of WT mice. Suspensions were labeled with PE-conjugated antimouse CD4 and CD4^+^ T cells enriched using the anti-PE magnetic cell sorting system (Miltenyi Biotec) according to manufacturer's instructions. Enriched CD4^+^ T cells (~85% pure, as estimated by flow cytometry) were then labeled with FITC-conjugated antimouse CD45RB and PE.Cy7-conjugated antimouse CD25. Naive T cells were sorted on a FACS Aria (Becton Dickinson) being CD4^+^CD25^−^CD45RB^high^ and >99% pure on reanalysis. Freshly generated BM-DC and sorted naive T cells were cocultured in 96-flat bottom plates in 200 *μ*L volume of complete IMDM, containing 1 × 10^4^ BM-DC and 2.5 × 10^4^ sorted naive CD4^+^ T cells, stimulated with immobilized anti-CD3 (clone 3C11, 5 *μ*g/mL) and soluble anti-CD28 (clone 37.51, 1 *μ*g/mL). Exogenous TGF*β* (5 ng/mL, R&D systems), and a given concentration of ST11, LPS, or LTA were added in order to get optimal T helper cell differentiation [24]. T helper cells differentiation was assessed after 4 days of coculture by flow cytometry analysis. Cells were stimulated 4 hours with PMA (50 ng/mL) and ionomycin (1 *μ*g/mL), treated with brefeldin A (5 *μ*g/mL) in the last two hours (all from Sigma), then harvested, cell surface stained with anti-CD4, then fixed and permeabilized with *Cytofix/Cytoperm* (Becton Dickinson), and intracellularly stained with anti-IL-17 and anti-IFN*γ*. Th1 cells were CD4^+^IFN*γ* ^+^ whereas Th17 cells were CD4^+^IL17^+^. Mesenteric lymph node cell suspensions were also made and treated as above to assess in adoptively transferred mice Th1 cells and Th17 cells *ex vivo*. Additionally, anti-FoxP3 staining was made in order to track the generation of so called CD4^+^FoxP3^+^ regulatory T cells. All antibodies were purchased from eBiosciences.

### 2.5. Colitis Induction and Morphological Assessment of Colonic Damages

To induce colitis, 5 × 10^5^ sorted naive CD4^+^ T cells in 200 *μ*L PBS were adoptively transferred i.p. into PBS- or ST11-fed Rag2^−/−^ mice then mice continued on their diet. Recipient mice were weighted initially and thereafter 3 times per week. Along the experiment, mice were observed for clinical signs of illness: hunched-over appearance, piloerection, diarrhea, and blood in the stool. In order to respect internal animal welfare policy (minimizing animal suffering), the regular protocol was shortened to avoid development of severe illness and mice were killed 4 weeks after T-cell transfer. No obvious signs of piloerection, diarrhea and blood in stools were observed within this period. Colonic tissue samples (systematically taken at mid-colon) were fixed in PBS containing 10% neutral-buffered formalin and paraffin embedded 5 *μ*m sections were made and stained with hematoxylin and eosin (H&E). The sections were analyzed under light microscopy without prior knowledge of the type of treatment.

### 2.6. Myeloperoxidase and Cytokine Measurements

Ultrasensitive multiplex cytokine profiling kit (Meso Scale Discovery) was used to assess mouse IL-1*β*, IL-6, KC (mouse IL-8), IL-10, IL-12p70, IFN*γ*, and TNF*α* in culture supernatants or whole colonic protein extracts according to manufacturer's instructions. IL-23 was measured in culture supernatants with standard ELISA kit (R&D systems). Myeloperoxidase (MPO) content of the colon protein extracts was determined with a mouse MPO ELISA kit (Hycult Biotech) following the manufacturer's instructions. When indicated cytokines or MPO levels were normalized to total tissue protein contents.

### 2.7. Total Protein and RNA Preparation

Total colon was cut longitudinally in two pieces snap frozen and stored at −80°C until protein and total RNA extraction were done respectively. Proteins from colon samples were prepared in RIPA buffer (Sigma) and protein measured with bicinchoninic acid assay kit (Thermo scientific). RNA preparation was made with RNADVANCE kit following manufacturer's instructions (AGENCOURT). Total RNA quality was assessed by Agilent RNA 6000 Nano LabChip Kit (Agilent Technologies) and quantification done with Ribogreen RNA Quantification Kit (Molecular Probes).

### 2.8. Real-Time PCR

Total RNA (1 *μ*g) was reverse-transcribed using Multiscribe Reverse Transcriptase (Applied Biosystems) following manufacturer's instructions. The cDNA was stored at −20°C until use. Then, 1 *μ*L of cDNA was mixed with 9 *μ*L of reaction mix containing 5 *μ*L 2X Syber Green Master Mix +3.6 *μ*L RNAse free Water +0.4 *μ*L Oligo Mix (11.35 *μ*M of forward and reverse primers) per well in a 384 well plates. Forward (fwd) and reverse (rev) primer sequences used for specific gene expression are available upon request. Real-time PCR was performed using the 7900 real-time PCR device (Applied Biosystem) with standard cycling conditions. The expression level of each gene is indicated by the number of cycles needed for the cDNA amplification to reach a threshold (Ct values). The Ct value for each gene was normalized to the housekeeping gene GAPDH. Relative mRNA expression value was calculated using the following formula: ΔCt = Ct of the gene of interest − Ct of GAPDH in the same sample, and mRNA relative expression was calculated based on the formula 2^−(ΔCt control)−(ΔCt colitis)^ for each sample.

### 2.9. Statistical Analysis

All the data presented herein are expressed as mean ± SD. Comparison between groups was made using student's *t*-tests for unpaired data when said or nonparametric Wilcoxon tests with the software SAS 9.1 when appropriate. Differences were considered statistically significant for *P* value <0.05.

## 3. Results

### 3.1. ST11 Mediated DC Activation and Helper T Cell Differentiation

**Figure 1:**
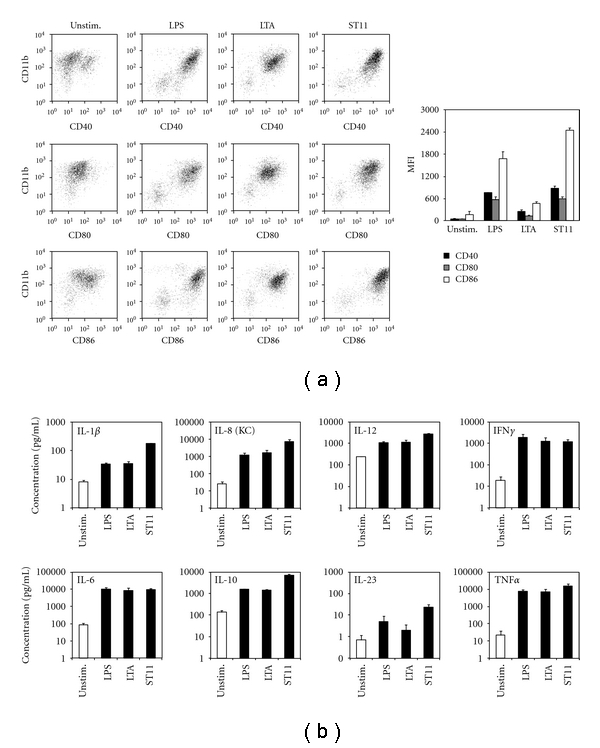
ST11 induces phenotypic DC maturation and specific cytokine release pattern. (a) 5 × 10^5^ BM-DCs were cultured for 24 h in the absence (Unstim.) or the presence of ST11 (at bacteria : DC ratio of 100 : 1). As positive control LPS or LTA stimulations were used at 1 *μ*g/mL. CD40, CD80, and CD86 expressions were determined by flow cytometry. Representative dot plots are shown in the left panel, and mean fluorescent intensity values ±SD for three separate experiments are shown in the right bar graph. (b) 5 × 10^5^ BM-DCs were cultured for 24 h in the absence (Unstim.) or the presence of ST11 (at bacteria : DC ratio of 100 : 1), LPS (0.1 *μ*g/mL), or LTA (1 *μ*g/mL), then supernatants were analyzed by multiplex ELISA. Results are mean values ±SD obtained in three independent cultures and expressed in pg/mL of supernatants for each condition.

The effect of ST11 on DC function was evaluated *in vitro* by analyzing maturation and cytokine production of immature BM-DCs exposed to different stimuli. BM-DCs were exposed 24 h with ST11 at ratio of 100 bacteria per cell. Results were compared to immature BM-DCs cultured in medium alone or exposed to LPS (0.1 *μ*g/mL) or LTA (1 *μ*g/mL) as positive control. Maturation pattern of BM-DCs was assessed by flow cytometry analysis of surface marker expression (Figure 1(a)) and ELISA determination of cytokine release in the culture supernatants (Figure 1(b)). As LPS and LTA, ST11 induced clear CD40, CD80, CD86 costimulatory molecules upregulation (Figure 1(a)). Next, we assess cytokine expression in supernatants. As LPS and LTA, ST11-mediated activation of BM-DC induce important secretion of proinflammatory cytokines (IL-1*β*, IL-6, IL-8, IL-12, IFN*γ*, and TNF*α*), as well as important amount of IL-10 which is an anti-inflammatory cytokine. Recently, anti-inflammatory probiotic properties were screened and distinguished according to their ability to preferentially induce IL-10 or IL-12 secretion, and strains leading to higher IL-10/IL-12 ratio were very potent in reducing TNBS-mediated intestinal inflammation [10, 25, 26]. Interestingly, ST11-activated BM-DCs made more IL-10 than IL-12 with an IL-10/IL-12 ratio of 2.91 ± 1.54.

**Figure 2:**
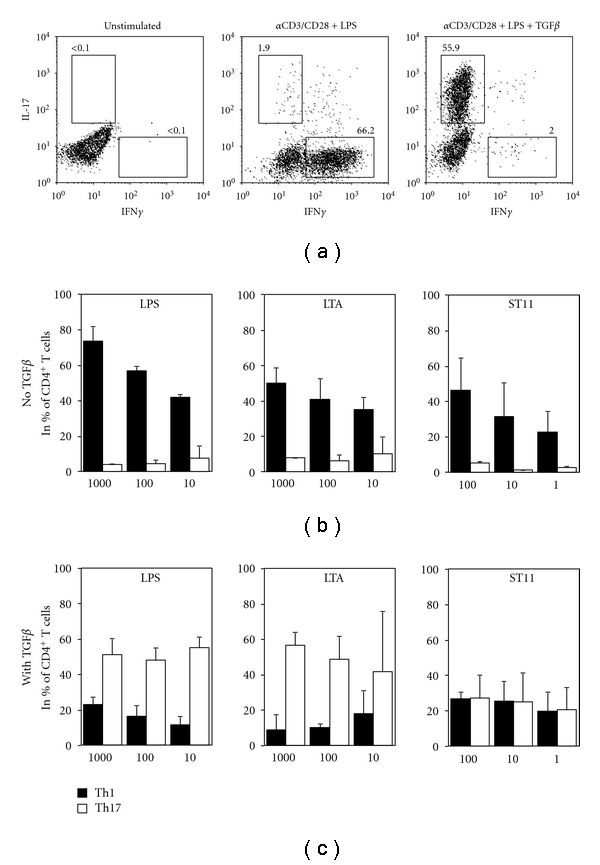
ST11 weakly supports Th17 cell differentiation in DCT cells cocultures. (a) BM-DCs were cocultured with sorted naïve CD4^+^ T cells for 4 days at DC : T cell ratio of 1 : 2.5 in the absence or the presence of TGF*β* (5 ng/mL). Then, cells were stimulated, fixed, and intracellularly stained for T helper 1 (Th1 being IFN*γ* ^+^) or T helper 17 (Th17 being IL-17^+^) cell quantification by flow cytometry. Representative dot-plots are shown, numbers indicate % of a gated population. Combination of proinflammatory signals and TGF*β* are mandatory for Th17 cell differentiation. (b) Bar graphs represent mean % values ±SD of Th1 (CD4^+^ IFN*γ* ^+^ IL-17^−^ T cells, black-filled bars) and Th17 (CD4^+^ IFN*γ* ^−^ IL-17^+^ T cells, unfilled bars) obtained in three independent experiments in the absence (a) or the presence (c) of TGF*β* and analyzed by flow cytometry. As proinflammatory signal, either LPS, LTA, or ST11 was used in a dose-dependent manner. Doses are given in ng/mL for LPS and LTA or bacteria: DC ratio for ST11.

As DC phenotype and cytokine expression tailor-specific immune responses *in vivo*, we evaluated whether ST11-mediated BM-DC activation support specific T cell polarizing activity. BM-DC and sorted naïve CD4^+^ T cell cocultures (DC-T) were made in the presence of different doses of LPS, LTA, or ST11. In classical coculture conditions (anti-CD3+ anti-CD28 stimulation without TGF*β*), almost exclusively IFN*γ* ^+^ producing T cells (so-called Th1 cells) were obtained (about 70%), whereas few if not any IL-17^+^ producing T cells (so-called Th17 cells) were found (<2%) (Figure 2(a), left panel). As recently described by Veldhoen and colleagues [24], TGF*β* introduction in T-DC coculture is necessary to initiate Th17 differentiation. Thus, by introducing TGF*β* into coculture, we restore *in vitro* Th17 differentiation (about 60%) and reduce Th1 differentiation (<2%) (Figure 2(a), right panel). Using the same protocol and different modulatory signals (i.e., LPS, LTA, or ST11) at different doses (ratio raging from 0.01–1 *μ*g/mL or 1 : 1 to 100 : 1 bacteria : cell), we studied how activated BM-DCs drive Th1 or Th17 differentiation. LPS and LTA-activated BM-DCs orchestrate in a dose-dependent manner Th1 differentiation in TGF*β*-free cocultures (Figure 2(b)) and Th17 differentiation in TGF*β*-containing cocultures (Figure 2(c)). Surprisingly, in clear contrast to LPS or LTA, ST11-activated BM-DCs were low Th1 inducers in TGF*β*-free cocultures (Figure 2(b)) and very poor Th17 inducers in TGF*β*-containing cocultures (Figure 2(c)) compared to LPS or LTA.

### 3.2. ST11-Fed Mice Are Protected against Colitis Induction

In our protocol, ST11 efficiently colonize GIT (data not shown) as previously described in mouse and human [18, 20] and displayed interesting *in vitro *immunomodulatory properties (above results), therefore we tested whether ST11 could be a potent anti-inflammatory strain *in vivo* allowing protection against intestinal damages associated with IBD. To test this hypothesis, we triggered colitis in rag2^−/−^ mice by adoptive transfer of sorted naïve T-cells. ECIBAT model of chronic intestinal inflammation was chosen because it induces a colonic inflammation which is closer to human IBD than other chemically-induced colitis models [27]. 

**Figure 3:**
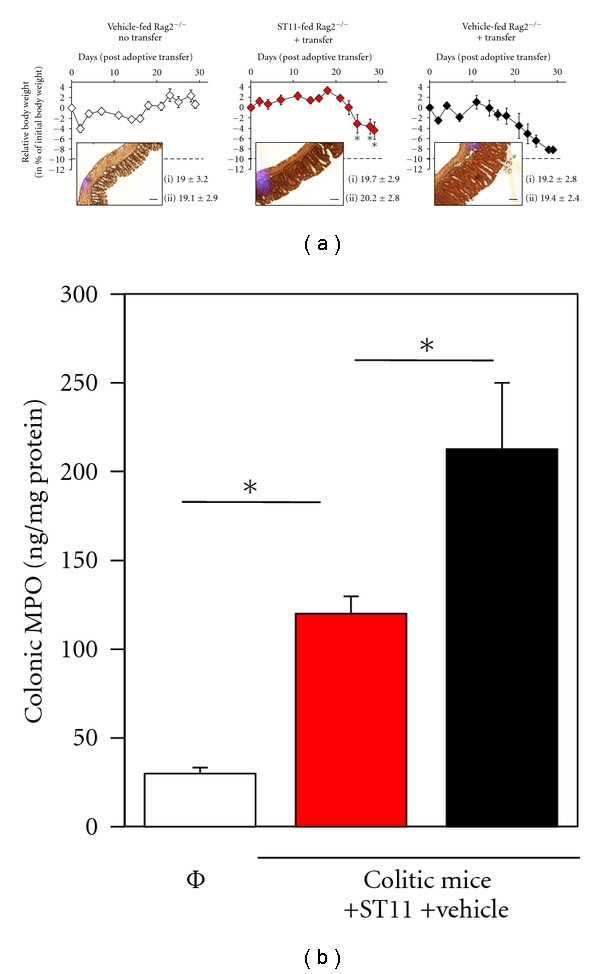
ST11 feeding inhibits colitis development induced by adoptive transfer of naïve T cell in Rag2^−/−^ mice. (a) 6–8 Rag2^−/−^ mice either PBS-fed (black diamonds, right graph) or ST11-fed (red diamonds, middle graph) were adoptively transferred with 5 × 10^5^ sorted naïve cells. Recipient mice were weighted 3 times a week. As negative control, a group of nontransferred Rag2^−/−^ mice was used (empty diamond, left graph). Weight changes (in % of initial body weight) of control or recipient mice ±SD are shown. Average weight in grams ±SD at the enrollment (i) and at day 0 before colitis induction (ii) is given for each group below the graphs. 4 weeks after cell transfer, mice were sacrificed and assessed for histopathology. Inserts are representative H&E stained pictures of colon sections; black bar represents 50 *μ*m (magnification ×20). (b) Colonic MPO was assessed in every single mouse by ELISA and expressed in ng MPO per mg of total colonic proteins. Student's *t*-test with *t*, tendency *P* < 0.1; *, *P* < 0.05, and **P < 0.01 versus PBS-fed mice.

ST11 feeding was not associated with significant difference in body weight before colitis induction as compared to vehicle PBS-fed mice (Figure 3(a) lower part). After colitis induction, ST11 feeding continues during 4 weeks posttransfer. Then, development of colitis was followed over time by measuring body weight, stool consistency, and general animal welfare. Marked and significant differences in body weight loss were observed around week 3 posttransfer between PBS-fed and ST11-fed colitic mice. We found that ST11 feeding was effective in protection against colitis as weight loss was significantly reduced by ~50% at week 4 post transfer compared to PBS-fed control mice with a mean of 3.6% versus 8.2% of initial body weight loss (Figure 3(a), black and red diamonds). In contrast, rag2^−/−^ healthy mice (PBS-fed, no T-cell transfer) slightly gain weight (Figure 3(a), empty diamonds). No other differences in macroscopic parameters were found between ST11-fed and PBS-fed mice. Specific colon inflammation provoked a twofold increase in weight/length (*W/L*) ratio in colitic mice compared to healthy mice but there was no marked effect of ST11 feeding. Notably, at 4 weeks post transfer, inflammation was localized to the colon and did not extend into the jejunum or ileum section of small intestine. Next, histological examination of H&E stained sections of colon at week 4 after colitis induction were made, some mucosal ulceration, hyperplasia and muscular thickening were equally observed in both PBS-fed and ST11-fed groups (Figure 3(a)). However, we observed a lesser neutrophil and mononuclear cell infiltrates in ST11-fed compared to PBS-fed colitic mice. As MPO is by far the most abundant protein product in azurophilic granules of neutrophils and is found in other polynuclear leukocytes, monocytes, and macrophages [28], we decided to measure MPO levels in order to unambiguously confirm our histological observations. We detected a significant reduction of colonic MPO level in ST11-fed compared to PBS-fed colitic mice (119.9 ± 9.8 versus 212.6 ± 37.3 ng/mg of total proteins); whereas in healthy mice, MPO levels measured were very low (29.9 ± 3.3 ng/mg of total proteins) (Figure 3(b)). All together, our results revealed that feeding ST11 strongly diminish development of colitis.

### 3.3. Immunological Features of ST11-Fed versus PBS-Fed Colitic Mice

In IBD patients or mouse models of IBD, colitis is primarily mediated by dysregulated mucosal DCs activation and pathogenic Th1 and Th17 effector T-cells generation [29–32]. Therefore, as a reflection of mucosal immune cell contents [33], we extensively analyzed mesenteric lymph node (MLN) cell phenotype and function in order to get insights into ST11-mediated protection. Based on *in vitro *results presented above, we pay attention to DC and T-cell subsets with a side by side comparison between healthy WT or colitic CD4^+^ T cells and DC phenotype and function. 

**Figure 4:**
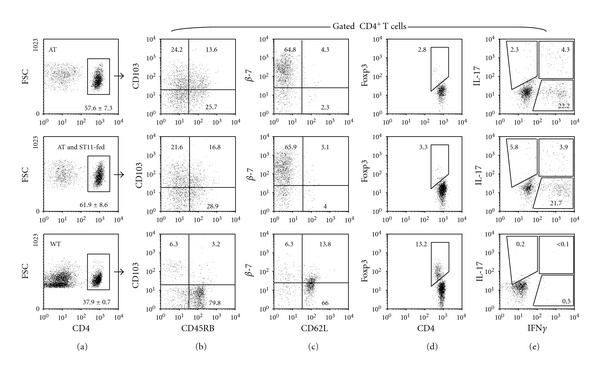
Phenotypic and functional characteristics of T cells after transfer. 5 × 10^5^ sorted naïve T cells were adoptively transferred into Rag2^−/−^ mice. (a) At week 4, mesenteric lymph node cell suspensions were made, stained, and analyzed by flow cytometry. CD4^+^ cells repopulated PBS-fed recipient mice (AT) as efficiently as ST11-fed mice (AT and ST11-fed). As control, representative staining obtained with CD4^+^ cells from unmanipulated C57BL/6 wild-type mice (WT) are shown (lower dot plots) because unmanipulated Rag2^−/−^mice do not contain CD4^+^ T cells. (b-d) Phenotype of gated CD4^+^ T cells was assessed by FoxP3, CD45RB, CD62L, CD103, and integrin *β*7 staining as well as (e) IL-17 and IFN*γ* secretion upon *in vitro* stimulation. Percentages are indicated in dot plots. Data are representative of two independent experiments made with 3-4 animals each time.

PBS-fed or ST11-fed rag2^−/−^ who received naïve T cells by adoptive transfer (AT) were efficiently colonized by CD4^+^ T cells. CD4^+^ T cells represent about 60% of live-gated lymphocytes in the MLN preparations. Non-T-cell-transferred rag2^−/−^ mice do not contain T cells, therefore wild-type (WT) mice were used as control and contained about 38% CD4^+^ T cells within the live-gated lymphocytes (Figure 4(a)). In colitic PBS-fed or ST11-fed rag2^−/−^ mice, T cells were the progeny of adoptively transferred T cells. Phenotypical characterization by flow cytometry supports their activated phenotype being CD45RB^low^ and CD62L^−^ in contrast to naïve CD45RB^high^ CD62L^+^ T cell inoculum. Transferred cells also express gut-homing classical markers CD103 and *β*7 integrin (Figures 4(b) and 4(c)). No numerical or phenotypical distinctions can be made between both PBS-fed and ST11-fed colitic mice. CD4^+^FoxP3^+^ regulatory T cells are known to suppress pathogenic effector cells through cell-to-cell contact or accumulation at the inflamed sites [31, 34–39]. Some probiotics have been shown to stimulate naïve T-cells conversion into Treg *in vitro* and *in vivo* and might at least partly explain probiotic immune functions [7, 12, 38]. Thus, we evaluated whether Treg cells were enriched in ST11-fed mice at mucosal inflammatory sites. FoxP3 staining did not reveal any significant difference in Treg cell numbers between PBS-fed and ST11-fed colitic mice. Indeed, all colitic mice contained about 3% Treg whereas healthy WT mice contained about 13% of CD4^+^FoxP3^+^ cells (Figure 4(d)). 

Additionally, T cells were stimulated *ex vivo* and IFN*γ* and IL-17 expression measured by flow cytometry. As expected, colitic mice were enriched in Th1 (CD4^+^IFN*γ* ^+^) and Th17 (CD4^+^IL17^+^) cells with about 22% and 2–5% respectively, whereas healthy WT mice contained few if not any of them (<0.5%) (Figure 4(e)). However, no significant differences can be found between PBS-fed and ST11-fed colitic animals. All together, these results ruled out a general ST11-mediated alleviation of inflammation by limiting gut homing, expansion, activation, and/or differentiation of transferred naïve T cells into lymphopenic recipients. 

**Figure 5:**
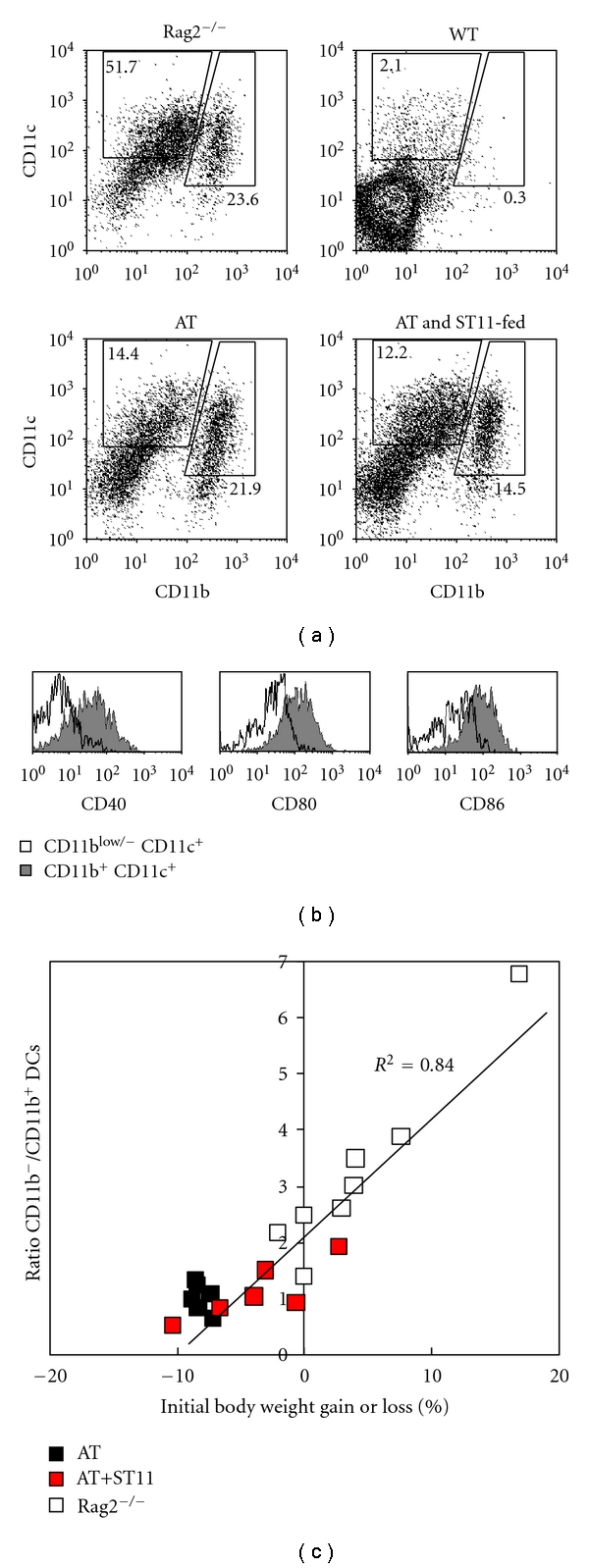
Phenotypic characterization of mesenteric DC after colitis induction. 5 × 10^5^ sorted naïve T cells were adoptively transferred into Rag2^−/−^ mice. (a) After 4 weeks, mesenteric lymph node cell suspensions were made, stained with anti-CD11c and anti-CD11b mAbs, and analyzed by flow cytometry. DC populations were equally important in colitic PBS-fed recipient mice (AT) and ST11-fed mice (AT and ST11-fed). As control, representative stainings obtained with unmanipulated C57BL/6 wild-type (WT) or Rag2^−/−^ mice (Rag2^−/−^) are shown. Among DCs, two different subsets were obtained: CD11b^−/low^CD11c^+^ and CD11b^+^CD11c^+^ DCs. (b) Representative staining showing CD40, CD80, and CD86 expressions on gated CD11b^−/low^CD11c^+^ (empty histograms) or CD11b^+^CD11c^+^ DCs (filled histograms) are shown. (c) Shown is a correlation curve between ratio of CD11b^−/low^CD11c^+^ and CD11b^+^CD11c^+^ DCs and body weight change in % loss (negative values) or gain (positive values) in colitic or healthy mice. Each symbol represents an individual mouse. Black-filled, red-filled, or empty squares were colitic PBS-fed mice, colitic ST11-fed mice, or healthy Rag2^−/−^ mice, respectively.

Next, we evaluated whether ST11-protective effects were associated to altered DC homeostasis in colitic mice. Both in human IBD patients and murine colitis models, pathology is associated with the local accumulation of activated DCs, that is, alterations in mucosal DC subsets are thought to contribute to the effector pathways that lead to IBD as recently reviewed in [40]. In our colitic mice, we confirmed CD11c^+^ DC abundance (about 30% of live gated cells) in MLN preparations (Figure 5(a)), whereas CD11c^+^ DC population represented less than 2-3% of live gated lymphocytes in healthy WT mice. However, at this stage, no significant differences can be found between PBS-fed and ST11-fed colitic animals. Recently, it was shown that distinct functions are supported by different DC subsets in the gut where two populations of DCs were described that is, CD11c^+^CD11b^low/−^ and CD11c^+^CD11b^+^ [41, 42]. The former population was reported to be beneficial and to favor gut tropism by upregulation of *β*7 integrin and CCR9 on naïve T cells as well as Treg induction, whereas the latter population was reported to be detrimental by improving pathogenic Th1 and Th17 cell differentiation. Therefore, we quantified both CD11c^+^CD11b^low/−^ and CD11c^+^CD11b^+^ DC subsets. Indeed, CD11c^+^CD11b^+^ DCs were highly enriched in colitic mice (more than half of total DCs, Figure 5(a)) and display an activated phenotype with higher level of costimulatory CD40, CD80, and CD86 molecules compared to their CD11c^+^CD11b^low/−^ DC counterparts (Figure 5(b)). In order to check whether this impaired balance between CD11c^+^CD11b^low/−^ and CD11c^+^CD11b^+^ DC subsets has consequences in colitis, we plot CD11b^low/−^/CD11b^+^ DC ratio against relative weight loss in every single mice (Figure 5(c)). We found a relevant correlation between colitis severity and enrichment in CD11b^+^ DCs. Interestingly, ST11-fed mice display less distortion in DC subsets and suggest that ST11 reinforces or supports DC homeostasis (ratio of 3.24, 1.13, versus 1.03 for healthy, ST11-fed colitic and PBS-fed colitic mice, resp.). This might explain at least partly preventive effect of ST11 feeding.

### 3.4. ST11-Feeding Attenuates Proinflammatory Cytokines Expression in Colon

**Figure 6:**
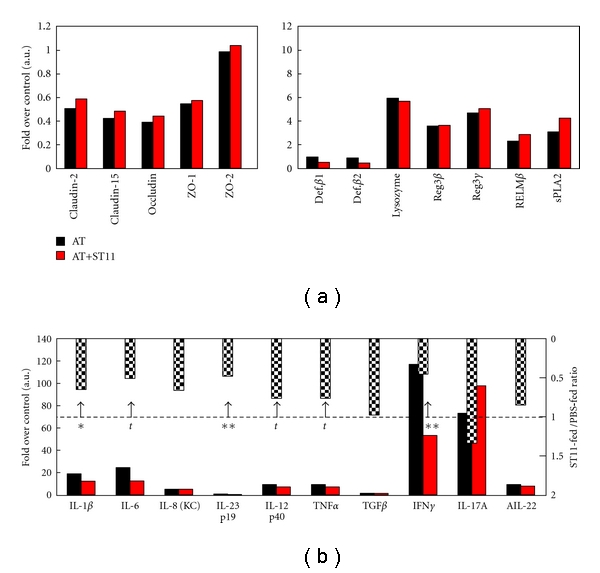
Gene expression analysis in colonic samples. Colonic mRNA expressions of tight junction proteins (Claudin-2, -15, occluding, ZO-1, and -2), antimicrobial peptides (defensin *β*-1, *β*-2, lysozyme, reg3*β*, reg3*γ*, RELM*β*, and secretory phospholipase A2), and cytokines (IL1*β*, IL-6, IL-8, IL-12, IL-23, TNF*α*, TGF*β*, IFN*γ*, IL-17A, IL17F, and IL-22) were evaluated in PBS-fed or ST11-fed colitic mice and given in the lower red and black bar graphs, respectively. GAPDH was used as a reference gene. Ratios between ST11-fed and PBS-fed mice are shown in the upper inverted bar graphs, respectively. For a given gene, mRNA expression is either decreased when the ratio <1 or increased when the ratio >1 in ST11-fed animals. *t*: tendancy *P* = 0.064*, *P* < 0.05, and **P < 0.01 versus PBS-fed mice.

**Figure 7:**
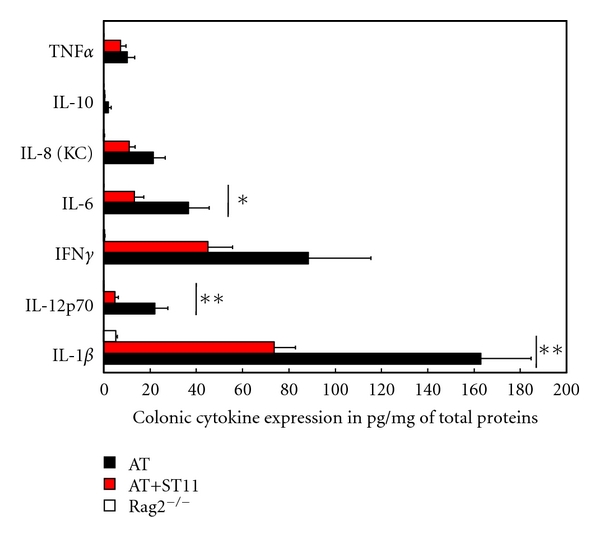
ST11 feeding reduces proinflammatory cytokine expression in colitic mice. Proinflammatory cytokine production by colonic tissues was assessed by multiplex ELISA at 4 weeks post transfer. Histograms show the mean value (in pg/mg of total proteins) ±SD (from 6–8 mice per group). Black-filled, red-filled, or empty bars were colitic PBS-fed mice (AT), colitic ST11-fed (AT+ST11), or healthy mice (Rag2^−/−^), respectively. *, *P* < 0.05, and **P < 0.01 versus PBS-fed mice.

To get more insights into how ST11 mediates its protective effect against colitis, further analyses were made on colonic samples. We evaluated the expression of several genes related to the gut immune system and inflammation. Gene-expression levels for cytokines, antimicrobial peptides and tight junction proteins were determined by real time quantitative PCR for each sample and normalized to the level of house-keeping GAPDH gene expression. Modulation of gut barrier function is frequently evoked in the literature to explain probiotic-associated health benefits [1, 15, 16]. Gut barrier function can be achieved by improving antibacterial peptide secretion and/or improving epithelial integrity. In order to test whether ST11-fed mice harbor better gut barrier condition, expression of mRNA for specific antibacterial peptides and tight junction proteins were quantified. Even though reduced or increased expressions were observed for anti-bacterial peptides or tight junction proteins mRNA respectively in colitic mice, none of the messenger assessed was significantly different between PBS-fed and ST11-fed colitic mice (Figure 6(a)). However, we observed a tendency or significant reduction of major proinflammatory cytokines mRNA levels for IL-1*β*, IL-6, IL-12, IL23, IFN*γ*, and TNF*α* (ST11-fed/PBS-fed ratio of relative gene expression <1, Figure 6(b), more importantly confirmed by significant reduction in colonic protein levels for IL-1*β*, IL-6, and IL-12 (Figure 7) in ST11-fed colitic mice compared to PBS-fed colitic mice. All together, these results suggest that ST11-mediated protection against colitis seems to be mediated by *in vivo* immunomodulatory properties of ST11 rather than gut barrier function modulation.

## 4. Discussion

Cellular and molecular effects of probiotics are actively studied, especially with respect to prevention and treatment of IBD [1, 15, 16]. Probiotics are by definition safe for human consumption, survive after ingestion within the GIT and, mediate strain specific health benefits. Their positive effects are related to microbiota modification, improvement gut barrier, and/or immunomodulatory properties. Herein, we studied specifically ST11-associated immune properties and health benefits. We showed that ST11 can strongly decrease colitis development in a preventive protocol. ST11 strain is of particular interest because its safety was already demonstrated, and it resists GIT conditions and is found in mouse stools as well as in human stools after feeding (data not shown and [18, 20]). Hence, we and others never observed adverse events in ST11-fed mice or human. 

Our *in vitro* studies improved current knowledge on ST11-specific immunomodulatory properties. ST11-activated BM-DCs made more anti-inflammatory IL-10 than proinflammatory IL-12 cytokines and were poor Th1/Th17 inducers. Many strains were tested and ST11 was one of the best IL-10 inducers and the weakest Th17 inducer (unpublished data). Molecular mechanisms behind these observations are still under investigations. Currently, we know that Th17 cells differentiate under the influence of DC-derived proinflammatory cytokines (IL-1*β*, IL-6, and IL-23) concomitantly with TGF*β* [24]. ST11-, LPS-, and LTA-activated BM-DC produced at least similar levels of IL-1*β* and IL-6, whereas IL23 was hardly detectable in all conditions in our hands. Therefore, differences in IL-1*β*, IL-6, or IL23 production by ST11-activated BM-DCs cannot be a reason to explain poor Th17 differentiation in T-DC coculture in presence of ST11. However, recently, it was shown that IL-10 reduces Th17 generation *in vitro* [43, 44], therefore we believe that reduction of Th17 differentiation with ST11-activated DC might be due to high level of IL-10 rather than the lack of a pro-Th17 cytokine. 

Our *in vivo* preventive protocol demonstrated that ST11 feeding was efficient in reducing colitis. In order to get more insights into mechanisms involved in ST11 health benefits on colitis, a careful flow cytometry study of mucosal regulatory T cells, helper T cells, and DCs in colitic mice was made. Treg generation is often associated with probiotic-mediated protection against inflammation [7, 12, 38]. In our study, ST11 neither improved Treg generation *in vitro* (data not shown) nor increased Treg numbers in colitic mice. We did not observe a significant reduction in effector T cells in ST11-fed compared to PBS-fed colitic mice, despite a weak Th1/Th17 generation potential *in vitro*. Moreover, mucosal DC recruitment and activation was studied based on recent evidence showing (i) probiotic feeding alters the distribution of the DC subsets within the intestinal lymphoid tissue [45] (ii) that impaired balance of pro-inflammatory CD11b^+^ and immunoregulatory CD11b^−^ mucosal DCs predisposes to colitis development [42]. Whereas ST11 feeding did not modify effector or regulatory T cell homeostasis in colitic mice, we observed an impact on DCs subset distribution. ST11 feeding limits CD11b^+^ pathogenic DCs recruitment and/or generation. CD11b^+^ pathogenic DCs abundance was clearly associated with disease severity (weight loss). It might be interesting to further characterize these cells and check whether ST11-feeding also affect DC homeostasis in noninflammed mice. 

No direct correlation can be made with some *in vitro* aspects obtained with ST11 culture and *in vivo* observations. This further supports that beyond primary *in vitro* screening, there is a need to assess probiotic activity in relevant complex physiological environment that reflects better the real life conditions and probiotic properties. According to literature evidences and our *in vitro* experiments, our prediction was that ST11 would protect against colitis based on reduction of Th1/Th17 pathogenic effector T-cells generation. To some extent this hypothesis was correct but ST11-mediated protection was not limited to reduction of pathogenic effector T-cells generation as reflected by our gene expression results in PBS-fed or ST11-fed inflamed colons. Following colitis induction, gut tight junction messengers were reduced, whereas antimicrobial peptide messengers were increased but independently of ST11 feeding. The increase of antimicrobial defenses might reflect the alteration of the gut barrier integrity initiating and/or sustaining intestinal inflammatory loop by enhanced luminal bacterial translocation. Improvement of gut barrier activity is a commonly used hypothesis to explain how probiotics exert their health benefits upon inflammatory challenge [1, 15, 16]. However, as ST11 feeding did not influence those gut barrier gene expression, we ruled out this hypothesis. When colitic mice were fed with ST11, an important and significant reduction of mucosal pro-inflammatory cytokines expression and neutrophils recruitment was observed. Thus, we believe that ST11 exerts a pivotal immunomodulatory role on innate cells which initially dampens global inflammatory responses *per se* thereby limiting subsequently intestinal tissue damages. 

All together, the results obtained in our preventive model of mouse colitis induction would favour a general reduction of intestinal innate immune response rather than any action on adaptive cell functions. We believed that ST11 mainly acts on innate cells such as DCs modifying their activity. However, as we only made a preventive study, we could not firmly exclude that, ST11 feeding reduces later helper T-cell generation or maintenance which in turn could also contribute to reduction of intestinal inflammation. Other mechanisms might participate to colitis reduction in probiotic fed animals. In ECIBAT mouse model of colitis, rapid and uncontrolled T-cell proliferation occurred in adoptively transferred immunodeficient mice [46]. Colitis reflects unregulated generation and activation of Th1 and Th17 that infiltrate the colon [29]. It might be driven by antigens from specific strain of enteric bacteria. Hence, IBD cannot be induced in immunodeficient mice reared under germ-free conditions, and major species of commensal bacteria are associated with IBD induction in different animal models [34, 47–49]. Of note, different helicobacter species were shown to be major IBD-elicitor strains [47–49]. Interestingly, we previously demonstrated that ST11 as well as *L. Johnsonii* were able to limit Helicobacter infection in human [21, 22, 50]. Therefore, probiotic-driven antagonism or out-competition of natural IBD eliciting flora might also be an interesting hypothesis to test in the future as a preventive approach to limit colitis onset.

Nutritional interventions, which are relatively easy to carry out, could have a larger impact on immune function than commonly appreciated. It is appealing to design probiotic-based preventive diet in order to maintain GIT homeostasis or prevent development of chronic inflammation. Herein, we show that ST11 probiotic strain harbors immune modulating properties* in vitro *and* in vivo *which reduce colitis*. *Therefore, we suggest that ST11 might be considered as a nutritional solution for IBD patients.
